# Rural–Urban Differences in Common Mental Disorders, Functional Limitation and Social Support among Adults with Cancer: A Population-Based Study in Spain

**DOI:** 10.3390/jcm11102742

**Published:** 2022-05-12

**Authors:** Silvia Portero de la Cruz, Jesús Cebrino

**Affiliations:** 1 Department of Nursing, Pharmacology and Physiotherapy, Faculty of Medicine and Nursing, University of Córdoba, Avda. Menéndez Pidal, S/N, 14071 Córdoba, Spain; n92pocrs@uco.es; 2 Research Group GC12 Clinical and Epidemiological Research in Primary Care, Instituto Maimónides de Investigación Biomédica de Córdoba (IMIBIC), Hospital Universitario Reina Sofía, 14071 Córdoba, Spain; 3 Department of Preventive Medicine and Public Health, Faculty of Medicine, University of Seville, Avda. Doctor Fedriani, S/N, 41009 Seville, Spain

**Keywords:** activities of daily living, cancer survivors, mental disorders, psycho-oncology, social support

## Abstract

The psychological, physical and social well-being components may differ for cancer patients living in urban vs. rural zones. This study aimed to examine the common mental disorders (CMDs), functional limitation (FL) and perceived social support (PSS) of rural and urban Spanish cancer patients from 2006 to 2017, to compare sociodemographic variables, health-related determinants and use of healthcare resources and to identify which sociodemographic and health-related factors were related to CMDs, FL and PSS. We performed a cross-sectional study among cancer patients using data from the Spanish National Health Surveys (2006, 2011 and 2017). A total of 698 of the subjects resided in rural areas and 1824 in urban areas. Binary logistic and multiple linear regressions were performed to determine the factors related to CMDs, FL and PSS. Rural residents visited their general practitioners more frequently than the city dwellers (61.03% vs. 56.63%, *p =* 0.04). A decreased prevalence of CMDs was observed among urban individuals over time (2006: 39.13%, 2011: 38.87%, 2017: 34.30%; *p* for trend = 0.04). Among rural residents, FL was associated with age, educational level and self-perceived health status, while among city dwellers, PSS was linked to marital status, nationality, having chronic conditions and self-perceived health status.

## 1. Introduction

The number of cancer survivors is rising worldwide, fueled by advances in early detection and treatment and the aging of the world’s population [1]. In Spain, between 2002–2007 and 2008–2013, the 5-year net survival rate increased by 3.3% (2002–2007: 52.0% and 2008–2013: 55.3%) for all cancers in men and by 2.6% overall for women (2002–2007: 59.1% and 2008–2013: 61.7%) [2].

Individuals with cancer must cope with the side effects of cancer treatment and how it affects their lives, leading to functional and cognitive impairments as well as other psychological and economic issues [3]. This has resulted in a growing interest in evaluating these aspects, which may vary in rural and urban areas, in cancer patients [4].

Regarding psychological functioning, cancer patients have an increased risk of mental health disorders compared with healthy populations [5]. Common mental disorders (CMDs), such as depression and anxiety, affect up to 20% and 10% of people with cancer, respectively [6]. CMDs in individuals with cancer can lead to negative consequences, including low treatment adherence, decreased survival rate and increased healthcare costs [7]. Although it has been suggested that urban living might be detrimental to the mental health of urban residents [8], current research on cancer patients suggests that living in a rural residence is related with decreased psychological well-being [9].

On the other hand, it is important to consider the influence of cancer on the ability to carry out daily activities. These activities of daily living are those that people perform in order to live independently within a society and can be divided into basic activities of daily life (e.g., washing or dressing) or more complex activities named as instrumental activities of daily living (e.g., shopping or using public transportation) [10]. Cancer has a significant impact on activities of daily living, as between 37% and 55% of cancer patients report having experienced difficulties or requiring assistance to complete activities of daily living [11]. Functional limitation (FL) in these activities influences decision making over treatments, as colorectal cancer patients who require assistance to perform at least two or more activities of daily living are 35% to 40% less likely to receive chemotherapy [12,13]. Moreover, higher mortality rates have been found in colorectal cancer individuals with two or more FLs [12]. Nevertheless, the functional status may be different in rural and urban areas. In this context, urban–rural differences have been reported in social and economic determinants of health, mental health issues and infrastructure and service delivery, with increased issues with transportation and providing healthcare services [14], all of which can affect the functional status of individuals with cancer.

Another essential factor to emphasize is well-being. In the case of cancer patients, a greater level of perceived social support (PSS), defined as the satisfaction gained by receiving assistance from members of a social network [15], is related with a higher experience of well-being [16] and plays an important role in coping with the disease and in improving quality of life [15]. PSS is also beneficial for the immune system in individuals with cancer, and those individuals with social support live approximately twice as long as those without such support [17]. A previous study has suggested that rural dwellers experience stronger social support than their urban counterparts [18].

Spanish studies have shown some factors affecting psychological, physical and social dimensions in cancer patients. For example, having a low socioeconomic status seems to have a clearly negative influence on physical, mental and social health in people with cancer [19]. In addition, there is evidence of a high prevalence of comorbid chronic conditions [20] and risk-promoting behavior, such as smoking, in Spanish cancer patients [21], all of which also have a negative influence on quality of life [22].

The evaluation of psychological, physical and social dimensions is relevant and should be part of the routine clinical analysis of cancer survivors at critical moments, such as receiving treatment and routine follow-up visits [23]. Taking this into account, it is important to examine these components according to place of residence, since they may differ in individuals with cancer living in rural and urban areas. Moreover, currently, there is very little research in Spain considering the geographical location. On the other hand, identifying the sociodemographic and health-related predictors involved is crucial, so that prompt, efficient interventions can be adapted or redesigned to take into account both these contributors and the residential location in order to improve quality of life in populations with cancer.

This study, which used a large, representative and national dataset of Spanish individuals with cancer, aimed to examine the presence of CMDs, FL and PSS in rural and urban Spanish individuals with cancer aged ≥18 years from 2006 to 2017. The objective was to compare sociodemographic variables, health-related determinants and use of healthcare resources in those individuals according to the residential location and to identify which sociodemographic and health-related factors were related to CMDs, FL and PSS among urban and rural individuals with cancer.

## 2. Materials and Methods

We conducted a cross-sectional study using individualized data from the Spanish National Health Surveys (SNHS) performed in 2006 [24], 2011 [25] and 2017 [26] and conducted by the Ministry of Health, Consumer Affairs and Social Welfare and the National Institute of Statistics.

### 2.1. Data Source

The SNHS are conducted on a representative sample of Spanish population. The sample approach used a multistage cluster, with the census as the primary unit, households as the secondary unit and household members as the tertiary unit. An adult from each household was randomly chosen to complete the survey and was mailed the reasons for the questionnaire, as well as the character and anonymity of participation. The interviews were conducted by an approved interviewer.

### 2.2. Study Participants

All people aged ≥18 years old were selected. Those who answered “yes” to the query, “Has your doctor told you that you are currently suffering from a malign tumor?” were identified as individuals with cancer. The sample initially comprised 2853 individuals with cancer (SNSH 2006: *n* = 965; SNSH 2011: *n* = 741; SNHS 2017: *n* = 1147), of whom 331 subjects (11.60%) were subsequently excluded due to refusal to answer the questions (SNSH 2006: *n* = 130; SNSH 2011: *n* = 109; SNHS 2017: *n* = 92), although their characteristics were similar to the other cancer subjects included. Finally, the total sample was composed of 2522 individuals: 835 in SNHS 2006; 632 in SNHS 2011; and 1055 in SNHS 2017. The sample was stratified, in terms of the rural/urban nature of their place of residence, by the number of inhabitants, as follows: rural towns (<10,000 inhabitants) and urban towns/cities (≥10,000 inhabitants) [27].

### 2.3. Study Variables

The variables were based on questions included in the questionnaires which were identical in all the surveys. The outcome variables in this study were the presence of CMDs, FL and PSS, which were assessed as follows.

CMDs. The 12-item General Health Questionnaire (GHQ-12) [28] validated in Spain [29,30] was used to identify them. The GHQ-12 has four possible answers, with the first two responses denoting the non-existence of a specific symptom (“0” points) and the last two revealing the existence of a symptom (“1” point) [31]. The total score ranges from 0 to 12 points, and the scores obtained for each item are added together. We set the cut-off point at ≥3 points, establishing the absence of CMDs by scores <3 points and their existence by scores ≥3 points, the latter being used to indicate the risk of psychological distress [31].

FL. This variable was investigated by using questions about different physical tasks in two functional domains [32,33]: (i) basic activities of daily life (e.g., personal hygiene or dressing); (ii) instrumental activities of daily life (e.g., food preparation or housekeeping). The difficulty level for each task was rated as: “I can do it by myself”, “I can do it with someone’s help” or “I am unable to do it at all”. When individuals replied “I can do it with someone’s help” or “I am unable to do it at all” in at least one item for a specific domain of FL, we identified the presence of FL.

PSS. This was measured using the Duke-UNC-11 questionnaire [34], validated in Spain [35,36]. This questionnaire includes 11 statements that assess the areas of social support as confident, emotional and instrumental support. It is scored using a 5-category Likert scale, with 1 being “far less than I would like” and 5 being “as much as I would like”. The larger the score, the greater the PSS. An overall score is calculated by summing all of the replies, which, in our study, ranged from 11 to 55 points.

The independent variables were classified into three groups: (i) sociodemographic variables, (ii) health-related determinants and (iii) use of healthcare resources.

Sociodemographic variables, including gender (men, women), age group (18–40 years, 41–64 years, ≥65 years), educational attainment (without studies, primary, secondary, professional training or university); marital status (single, married, widowed, separated/divorced), social class (upper: classes I and II, middle: classes III and IV, lower: classes V and VI) [37] and nationality (Spanish, foreign).

The health-related determinants consisted of body mass index (BMI) (underweight, normal weight, overweight and obese) [38], smoking status (yes, no), alcohol intake in the last year (yes, no), number of comorbid chronic conditions (none, one or two, and three or more) and self-perceived health status (very good, good, fair, poor, very poor).

Use of healthcare resources was evaluated through visits to the general practitioner in the previous 4 weeks (yes, no), visits to a specialist physician in the previous 4 weeks (yes, no), use of emergency services in the previous year (yes, no) and hospitalizations in the previous year (yes, no).

### 2.4. Procedure and Ethical Considerations

The anonymized data are available to the general public through the National Institute of Statistics and Ministry of Health, Consumer Affairs and Social Welfare sites [36,37,38]. According to Spanish law, authorization by an Ethics Committee was deemed unnecessary because a de-identified public database was used. The research data are included in the Supplementary File S1.

### 2.5. Statistical Analysis

Descriptive analysis was carried out using counts, percentages and mean and standard deviation. The Chi-squared test was used to compare proportions. Chi-square trend analysis and linear regression model were performed to recognize significant trends in the presence of CMDs, FL and PSS from 2006 to 2017. Subsequently, two multivariate logistic regressions were performed to find the factors associated with the presence of CMDs and FL. The measures of estimated association were the crude and adjusted odds ratio (OR) with confidence intervals set at 95%. The Hosmer–Lemeshow test was used to assess the goodness of fit. For PSS, we fitted a multiple linear regression model. We examined the adjusted coefficient of determination (R^2^), the F statistic and the normality of the residues to determine the goodness of fit. For the multivariate models, we only included the variables that had a potential association with each dependent variable (*p* ≤ 0.15), and backward selection was used to eliminate non-significant variables based on the likelihood of the Wald statistic. For purposes of the multivariate analysis, the variables were reorganized as follows: age group (<65 years old, ≥65 years old), educational attainment (without/primary studies, secondary/university studies), marital status (married, non-married), BMI (normal weight, non-normal weight), chronic conditions (yes, no), self-perceived health status (very good/good, fair, very poor/poor). Statistical significance was set at α = 0.05. The IBM SPSS Statistical package version 25 (IBM Corp, Armonk, NY, USA) was used for the statistical analysis, which was licensed to the University of Cordoba (Córdoba, Spain).

## 3. Results

### 3.1. Sociodemographic Variables, Health-Related Determinants and Use of Healthcare Resources

Of the cancer individuals identified, 27.68% (*n* = 698) resided in areas defined as rural and 72.32% (*n* = 1824) in urban areas. Statistically significant differences in sociodemographic characteristics, health-related determinants and use of healthcare resources between rural and urban individuals were observed. Rural residents reported a lower prevalence of cancer (*p* < 0.0001), were older (*p* = 0.001), had a lower educational level (*p* < 0.001), belonged to a lower social class (*p* < 0.001) and reported a less common use of tobacco (*p* < 0.01) and alcohol (*p* < 0.001) than urban participants (Table 1).

**Figure 1:**
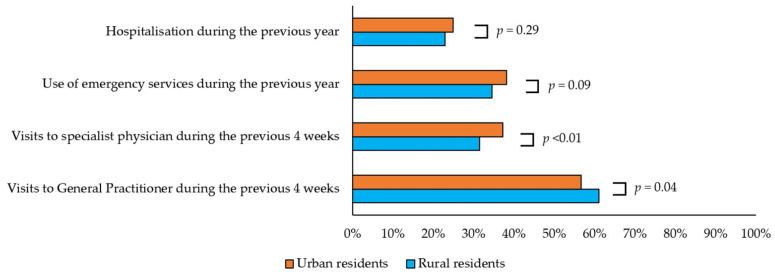
Use of healthcare resources by cancer patients, according to place of residence.

Moreover, we observed an increase in the prevalence of cancer patients between both groups (rural: 2006: 2.81%, 2011: 2.07%, 2017: 4.38%, *p* for trend <0.0001; urban: 2006: 2.92%, 2011: 4.45%, 2017: 4.95%, *p* for trend < 0.0001). Figure 1 shows the use of healthcare resources by the rural and urban populations. Although the rural participants used emergency services and were hospitalized about as frequently as urban residents (*p* = 0.09 and *p* = 0.29, respectively), rural residents saw their general practitioners more often than urban participants (*p* = 0.04). On the other hand, urban participants visited a specialist physician more frequently than rural residents (*p* < 0.01).

### 3.2. CMDs, FL and PSS

With regard to CMDs, we found no differences between rural and urban residents (37.11% vs. 36.95%, respectively; *p* = 0.94). Nevertheless, over the study years, the prevalence of CMDs among urban cancer patients decreased (2006: 39.13%, 2011: 38.87%, 2017: 34.30%; *p* for trend = 0.04) but not among rural residents (2006: 40.28%, 2011: 35.90%, 2017: 34.36%; *p* for trend = 0.16). In relation to FL, rural participants reported a significantly higher prevalence compared to urban residents (35.24% vs. 30.15%, respectively; *p* = 0.01). Moreover, we discovered an increase in the prevalence of FL between both groups (rural: 2006: 30.74%, 2011: 27.56%, 2017: 44.79%, *p* for trend <0.001; urban: 2006: 15.76%, 2011: 22.06%, 2017: 44.97%, *p* for trend <0.001). On the other hand, there were no significant associations between PSS and rural–urban place of residence (47.93 ± 8.40 points vs. 47.25 ± 8.31 points; *p* = 0.07). No significant changes were observed in either rural or urban residents over the study period in relation to PSS (rural: 2006: 47.47 ± 9.29 points, 2011: 48.06 ± 8.52 points, 2017: 48.35 ± 7.22 points, *p* for trend = 0.40; urban: 2006: 47.42 ± 8.82 points, 2011: 47.29 ± 8.61 points, 2017: 47.11 ± 7.76 points, *p* for trend = 0.50).

### 3.3. Associations between Sociodemographic Variables, Health-Related Determinants, CMDs, FL and PSS

Table 2 shows the results from logistic regression models of CMDs in rural and urban participants. In rural residents with chronic conditions or a fair or very poor/poor self-perceived health status, the probability of suffering from CMDs was higher. In urban residents, having consumed alcohol in the last year and visiting a specialist physician decreased that probability.

The results from the logistic regression models of FL in rural and urban participants are shown in Table 3. Three variables were found to be related with a greater probability of FL in rural residents: being ≥ 65 years old, having no studies or primary studies and having a fair or very poor/poor self-perceived health status. In urban participants, the probability of FL was higher in those who were ≥ 65 years old, had no studies or primary studies, were Spanish, had non-normal weight and had a fair or very poor/poor self-perceived health status.

Table 4 shows the multiple linear regression models considering social support as a dependent variable and sociodemographic and health-related factors as independent variables, according to the place of residence. Among the participants who lived in rural areas, PSS was positively related with being married and negatively with smoking and having a fair or very poor/poor self-perceived health status. In urban residents, being married, being Spanish and having chronic conditions were positively associated with the PSS.

## 4. Discussion

### 4.1. Sociodemographic Characteristics, Health-Related Determinants and Use of Healthcare Resources: Differences across Places of Residence

The findings of the current study show that rural participants were older, had a lower educational level, belonged to a lower social class and reported a less common use of tobacco and alcohol than their urban counterparts. On the other hand, cancer diagnosis was more prevalent among urban participants, which could be due to the fact that urban dwellers are subjected to higher levels of industrial pollution than rural residents, and they are more vulnerable to important cancer risk factors, such as overweight and obesity, cigarette smoking, alcohol use and physical inactivity and are more likely to participate in cancer screening [39]. Although universal health coverage has eliminated many barriers to receiving suitable, high-quality health care, in the present study, geographic location seems to influence the type of healthcare services used by cancer patients. In our case, the participants from rural areas reported a higher use of primary care and a lower use of specialist care than urban residents. Specialists and subspecialists generally tend to be concentrated in urban areas, resulting in a greater reliance on primary care providers in rural areas [40]. Moreover, the greater use of primary care by people residing in rural areas may be due to a lack of willingness or because of transportation issues [41]. Taking this into account, general practitioners should be trained to care for cancer patients, and communication with oncologists should be encouraged early on in the training [42].

### 4.2. CMDs, FL and PSS

The fact that mental health is affected systematically according to the characteristics of the place of residence or the neighborhood has been reported [43]. Living in cities is associated with specific environmental factors, such as high levels of air pollution and lack of access to green areas [44]. In Spain, the Spanish Healthy Cities Network [45], which is a sub-section of the Spanish Federation of Municipalities and Provinces and complies with the principles of action set out in the World Health Organization’s “Healthy Cities” project [46], provides measures, which can contribute toward mitigating mental health issues, such as increased physical exercise. This is achieved through policies that influence the forms of active transport and the availability of spaces for doing physical activity and creating green areas, which can positively reduce exposure to air pollution, noise and heat and facilitate social cohesion. These measures could have contributed to the reduced prevalence of CMDs seen in the present study among urban dwellers from 2006 to 2017 despite the prevalence of cancer patients increasing over the same years in our study.

Taking into account that, in the current study, the percentage of cancer diagnosis was higher among urban participants, cancer patients in rural areas tended to have higher rates of FL than urban dwellers, although the prevalence of FL increased from 2006 to 2017 in both groups. This is in line with another study [47]. Compared with the urban residents, people with FL in rural areas may face additional barriers (transportation problems or difficult access to education and health care). These challenges, together with shifts in labor market conditions and existing health disparities, may have also contributed to the progression of FL [48].

### 4.3. Sociodemographic Characteristics and Health-Related Factors Associated with CMDs in Rural and Urban Cancer Patients

The current results revealed that alcohol intake and a fair or poor/very poor self-perceived health status were related with a lower risk of CMDs in rural and urban residents. Regarding alcohol consumption, researchers have noted that cancer patients may use this substance in attempts to cope with psychological distress [49]. In relation to self-perceived health status, our findings are in line with the previous literature [50]. Self-perceived health status is widely considered a valid measure of general health [49]. The relationship between self-perceived health status and CMDs obtained in the current study required more in-depth investigation, as CMDs are not necessarily a direct outcome of fair or poor/very poor health status but can also be a predictor [51]. Further longitudinal studies are needed to establish this association. In our study, having chronic conditions increased the probability of suffering from CMDs in rural participants. The presence of a chronic condition together with limited healthcare access and limited insurance and transportation issues may affect perceived life satisfaction more strongly and consistently, which in turn would have a greater effect on mental health [52]. 

On the other hand, socioeconomic status has been widely proposed as a predictor of specific cancer outcomes among cancer patients [53]. A recent study [54] found that low socioeconomic status is negatively associated with mental health and self-rated health. In the current study, having no studies or primary studies and belonging to middle or lower social class increased the probability of suffering from CMDs in participants residing in urban areas. This result can be explained by the fact that individuals with a low socioeconomic status tend to perceive their neighborhood as unsafe, which results in worse mental health outcomes [55].

With regard to gender, women who lived in urban areas were more likely to experience CMDs. Among female cancer patients, certain symptoms of CMDs, such as depression and anxiety, are generally found more frequently than in men [56], and women are also more likely to express the need for psychosocial support of their own accord [57]. In urban areas, it has been reported that women establish more neighborhood ties and have a higher attachment to the community than men.

### 4.4. Sociodemographic Characteristics and Health-Related Factors Associated with FL in Rural and Urban Cancer Patients

In the current study, being ≥65 years old and having a worse self-perceived health status were associated with a higher probability of FL for cancer patients in urban and rural areas. Older adults with cancer not only suffer from cancer-generated health problems but also encounter other age-related medical conditions, which have been associated with increased FLs [58]. According to the findings of this study, previous research has reported that people with poor self-perceived health are at a high risk for FL in the non-cancer population [59]. Furthermore, it is believed that a worse self-perceived health is associated with inflammatory biomarkers, including C-reactive protein and cytokines, which may contribute to FL [60].

In the present population-based study, and in line with a previous study [61], people with low educational attainment residing in both urban and rural areas had a higher probability of FL. It seems that individuals with a higher educational level tend to follow a healthy lifestyle and seek out medical knowledge to avoid FL across different contexts [62].

In relation to marital status, our findings show that, among married urban cancer patients, the probability of FL decreased. Research has shown that among those individuals who are married, those with a higher-quality marriage have better physical health and self-rated health [63].

On the other hand, the results of the current study showed that the relation between FL and BMI varied among urban and rural groups. Here, the findings showed a significantly higher probability of FL in cancer patients with non-normal BMI living in urban districts, while this relationship was not observed for rural participants. This was partly consistent with a previous study performed in non-cancer populations [64]. One possible reason is the existence of inequalities between rural and urban individuals’ access to healthcare services in Spain [65].

In our study, the probability of FL was lower among foreign cancer patients living in urban areas. The evidence suggests that the foreign population has better health and lives longer than their native counterparts because they have healthy characteristics, which enabled them to immigrate in the first place [66].

### 4.5. Sociodemographic Characteristics and Health-Related Factors Associated with PSS in Rural and Urban Cancer Patients

In the current study, PSS was positively associated with married individuals residing in rural and urban areas. Marriage provides structural and functional support, resulting in an overall biological, psychological and economic advantage that may make these cancer patients more health aware [66]. On the other hand, according to previous evidence [67], the significant association between low PSS and a fair or poor/very poor self-perceived health status seems to suggest that these negative interactions with members of their local network might be an upsetting stressor.

Compared to their urban counterparts, rural dwellers are more likely to engage in health-risk behaviors, such as tobacco use [68]. In the current study, PSS was negatively associated with smoking. The existing research suggests that social networks can exert a powerful influence on health; however, evidence from rural contexts is limited [69]. According to current smokers among the oncological population, the obstacles to them quitting smoking include those close to them smoking, the sense of inadequate support as a result of critical remarks about previous unsuccessful attempts to quit and gloomy statements about future attempts to stop [70].

The results of the current study showed that having more comorbid medical conditions and residing in urban areas was positively related to PSS. This may be due to the fact that people with chronic conditions require more social support than individuals without chronic diseases because of the added effect of the stressors and self-management requirements of each disease [71]. As for nationality, being an urban Spanish resident was positively associated with PSS. Migration, which results in a loss of social networks and social exclusion in the new country, contributes to a lack of social support, all of which makes foreigners more likely to report a poor self-perceived health status [72]. Interventions that facilitate foreign people’s social networking could be one way of increasing their social support, facilitating integration, reducing feelings of loneliness, and thereby preventing poor health.

### 4.6. Strengths and Limitations

This study includes both strengths and limitations. The large sample size, the fact that the information was obtained using a consistent methodology across time and the huge number of sociodemographic and health variables collected are all positives. Nonetheless, we should also point out some of our study’s limitations: (i) the questions about the presence of a malign tumor were not validated, and medical records were not requested; (ii) information obtained through an interview may be prone to memory or social desirability biases; (iii) because our study only included variables from the Spanish National Health Surveys, it was not possible to add other clinical or cancer-related variables; and (iv) the causality in the associations found cannot be established due to the cross-sectional design.

### 4.7. Implications for Research and Practice

Our findings further contribute to the body of research supporting the relevance of taking into account the location of residence and its influence on the physical, psychological and social dimensions of cancer patients. Given our findings, future studies need to explore coping abilities and resilience in cancer patients in both urban and rural settings. The findings of this study highlight the need for continuing to give supportive care to rural cancer patients to enhance their well-being. Moreover, as the number of cancer patients grows, it is essential to plan clinical and policy interventions to eliminate inequities in physical, psychological and social status in rural vs. urban regions.

## 5. Conclusions

The sociodemographic characteristics and health-related factors that vary according to the place of residence are: prevalence of cancer patients, age, educational level, social class and consumption of tobacco and alcohol. With regard to the use of healthcare resources, rural residents see their general practitioners more frequently than urban participants, and the latter visit specialists more frequently than rural residents. On the other hand, this study shows an increased prevalence of cancer patients from 2006 to 2017 in rural and urban areas, as well as a decreased prevalence of CMDs among urban individuals over time. The prevalence of FL is higher in rural participants, although the number of people with cancer who have FL increased from 2006 to 2017 between both rural and urban participants. Among rural residents, CMDs are associated with alcohol consumption, the presence of chronic conditions, visits to the general practitioner and self-perceived health status, while in urban participants, they are linked to gender, educational level, social class, alcohol consumption, visits to a specialist physician and self-perceived health status. The presence of FLs in rural individuals is related to age group, educational level and self-perceived health status, while in urban residents, it is associated with age group, educational level, marital status, nationality, BMI, use of emergency services and self-perceived health status. Finally, PSS is linked to marital status, tobacco consumption and self-perceived health status in rural participants, while it is associated with marital status, nationality, the presence of chronic conditions and self-perceived health status among urban residents.

## Figures and Tables

**Table 1:** Sociodemographic characteristics and health-related determinants of rural and urban cancer individuals (*n* = 2522).

Variables	Total *n* (%)	Rural *n* = 698 (%)	Urban *n* = 1824 (%)	*p*-Value
Gender				0.41
Men	961 (38.10)	275 (39.40)	686 (37.61)	
Women	1561 (61.90)	423 (60.60)	1138 (62.39)	
Age group				0.001
18–40 years old	124 (4.92)	33 (4.72)	91 (4.99)	
41–64 years old	1023 (40.56)	244 (34.96)	779 (42.71)	
≥65 years old	1375 (54.52)	421 (60.32)	954 (52.30)	
Educational attainment				<0.001
Without studies	512 (20.30)	196 (28.08)	316 (17.32)	
Primary	773 (30.65)	262 (37.54)	511 (28.02)	
Secondary or PT	922 (36.56)	198 (28.37)	724 (39.69)	
University	315 (12.49)	42 (6.01)	273 (14.97)	
Marital status				0.17
Single	298 (11.82)	88 (12.61)	210 (11.51)	
Married	1524 (60.43)	421 (60.32)	1103 (60.47)	
Widowed	511 (20.26)	149 (21.35)	362 (19.85)	
Separated or divorced	189 (7.49)	40 (5.72)	149 (8.17)	
Social class				<0.001
Upper	452 (17.92)	79 (11.32)	373 (20.45)	
Middle	1084 (42.98)	298 (42.69)	786 (43.09)	
Lower	986 (39.10)	321 (45.99)	665 (36.46)	
Nationality				0.37
Spanish	2472 (98.02)	687 (98.42)	1785 (97.86)	
Foreigner	50 (1.98)	11 (1.58)	39 (2.14)	
Body Mass Index				0.28
Underweight	45 (1.78)	16 (2.29)	29 (1.59)	
Normal weight	942 (37.35)	272 (38.97)	670 (36.73)	
Overweight	990 (39.25)	273 (39.11)	717 (39.31)	
Obesity	545 (21.62)	137 (19.63)	408 (22.37)	
Smoking status				<0.01
Yes	380 (15.07)	81 (11.60)	299 (16.39)	
No	2142 (84.93)	617 (88.40)	1525 (83.61)	
Alcohol intake in the last year				<0.001
Yes	931 (36.92)	204 (29.23)	727 (39.86)	
No	1591 (63.08)	494 (70.77)	1097 (60.14)	
Number of chronic conditions				0.32
0	379 (15.03)	102 (14.62)	277 (15.19)	
1–2	732 (29.02)	218 (31.23)	514 (28.18)	
≥3	1411 (55.95)	378 (54.15)	1033 (56.63)	
Self-perceived health status				0.54
Very good	103 (4.09)	22 (3.16)	81 (4.44)	
Good	726 (28.79)	194 (27.79)	532 (29.17)	
Fair	962 (38.14)	273 (39.11)	689 (37.77)	
Poor	533 (21.13)	150 (21.49)	383 (21.00)	
Very poor	198 (7.85)	59 (8.45)	139 (7.62)	

PT: Professional Training.

**Table 2:** Association between CMDs, sociodemographic factors and health-related determinants in rural and urban cancer patients (*n* = 2522).

Variables	Rural Residents			Urban Residents		
	OR (IC 95%)	ORa (IC 95%)	*p*-Value	OR (IC 95%)	ORa (IC95%)	*p*-Value
Gender						
Men	Reference			Reference	Reference	
Women	1.17 (0.85–1.60)			1.40 (1.14–1.70)	1.37 (1.09–1.71)	0.01
Age group						
<65 years old	Reference			Reference		
≥65 years old	1.05 (0.77–1.43)			0.87 (0.72–1.06)		
Educational attainment						
Without/Primary studies	1.36 (0.98–1.89)			1.31 (1.08–1.58)	1.17 (1.11–1.28)	0.001
Secondary/University studies	Reference			Reference	Reference	
Marital status						
Married	1.02 (0.75–1.40)			0.83 (0.68–1.12)		
Non-married	Reference			Reference		
Social class						
Upper	Reference			Reference	Reference	
Middle	1.47 (0.86–2.51)			1.85 (1.40–2.44)	1.65 (1.21–2.26)	0.001
Lower	1.53 (0.90–2.62)			2.38 (1.80–3.16)	1.98 (1.44–2.76)	<0.001
Nationality						
Spanish	1.03 (0.30–3.56)			1.50 (0.74–3.04)		
Foreigner	Reference			Reference		
Body Mass Index						
Normal weight	Reference			Reference		
Non-normal weight	0.98 (0.73–1.37)			1.14 (0.93–1.39)		
Hospitalization during the previous year						
Yes	0.87 (0.76–1.53)			0.44 (0.35–0.54)		
No	Reference			Reference		
Use of emergency services during the previous year						
Yes	0.65 (0.55–1.14)			0.42 (0.35–0.51)		
No	Reference			Reference		
Visits to specialist physician during the previous 4 weeks						
Yes	0.49 (0.36–0.68)			0.43 (0.36–0.53)	0.62 (0.50–0.77)	<0.001
No	Reference			Reference	Reference	
Visits to general practitioner during the previous 4 weeks						
Yes	0.47 (0.34–0.66)	0.74 (0.51–0.91)	0.03	0.53 (0.43–0.64)		
No	Reference	Reference		Reference		
Smoking status						
Yes	1.06 (0.66–1.70)			1.18 (0.91–1.51)		
No	Reference			Reference		
Alcohol intake in the last year						
Yes	0.58 (0.41–0.82)	0.67 (0.46–0.97)	0.04	0.53 (0.44–0.65)	0.69 (0.55–0.87)	<0.001
No	Reference	Reference		Reference	Reference	
Chronic conditions						
Yes	1.99 (1.23–3.22)	1.52 (1.10–2.61)	0.01	1.31 (0.99–1.72)		
No	Reference	Reference		Reference		
Self-perceived health status						
Very good/good	Reference	Reference		Reference	Reference	
Fair	2.30 (1.48–3.56)	1.98 (1.26–3.11)	0.001	2.88 (2.21–3.76)	2.57 (1.95–3.37)	<0.001
Very poor/poor	3.51 (2.02–4.03)	5.85 (3.82–6.15)	<0.001	4.94 (3.02–5.91)	4.43 (4.31–5.10)	<0.001

OR: odds ratio; ORa: odds ratio adjusted for all sociodemographic and health-related variables; CI 95%: 95% confidence interval; Rural residents: Hosmer–Lemeshow test for CMDs χ^2^ = 3.74, *p* = 0.61; Nagelkerke’s R^2^ Square for CMDs = 0.19; *p*-value < 0.001; Urban residents: Hosmer–Lemeshow test for CMDs χ^2^ = 5.21, *p* = 0.76; Nagelkerke’s R^2^ Square for CMDs = 0.21; *p*-value < 0.001.

**Table 3:** Association between FL, sociodemographic factors and health-related determinants in rural and urban cancer patients (*n* = 2522).

Variables	Rural Residents			Urban Residents		
	OR (IC 95%)	ORa (IC 95%)	*p*-Value	OR (IC 95%)	ORa (IC 95%)	*p*-Value
Gender						
Men	Reference			Reference		
Women	0.81 (0.59–1.11)			1.02 (0.83–1.26)		
Age group						
<65 years old	Reference	Reference		Reference	Reference	
≥65 years old	9.10 (5.92–9.98)	8.41 (5.21–9.40)	<0.001	9.60 (7.37–9.96)	9.04 (6.75–9.49)	<0.001
Educational attainment						
Without/Primary studies	3.65 (2.50–5.32)	1.60 (1.03–2.50)	0.04	2.31 (2.25–3.39)	1.43 (1.11–1.84)	<0.01
Secondary/University studies	Reference	Reference		Reference	Reference	
Marital status						
Married	0.97 (0.70–1.33)			0.59 (0.48–0.73)	0.67 (0.52–0.85)	0.001
Non-married	Reference			Reference	Reference	
Social class						
Upper	Reference			Reference		
Middle	1.55 (0.88–2.74)			1.32 (0.99–1.76)		
Lower	2.12 (1.21–3.72)			1.98 (1.48–2.64)		
Nationality						
Spanish	1.46 (0.38–5.55)			5.30 (1.63–5.29)	3.87 (1.07–4.02)	0.04
Foreigner	Reference			Reference	Reference	
Body Mass Index						
Normal weight	Reference			Reference	Reference	
Non-normal weight	1.02 (0.74–1.41)			1.93 (1.55–2.41)	1.49 (1.14–1.94)	<0.01
Hospitalization during the previous year						
Yes	0.63 (0.44–0.90)			0.68 (0.54–0.85)		
No	Reference			Reference		
Use of emergency services during the previous year						
Yes	0.41 (0.29–0.56)			0.47 (0.39–0.58)	0.60 (0.47–0.77)	<0.001
No	Reference			Reference	Reference	
Visits to specialist physician during the previous 4 weeks						
Yes	0.75 (0.54–1.06)			0.82 (0.67–1.10)		
No	Reference			Reference		
Visits to general practitioner during the previous 4 weeks						
Yes	0.62 (0.45–0.86)			0.80 (0.66–0.98)		
No	Reference			Reference		
Smoking status						
Yes	0.26 (0.13–0.49)			0.43 (0.32–0.60)		
No	Reference			Reference		
Alcohol intake in the last year						
Yes	0.57 (0.40–0.81)			0.90 (0.74–1.11)		
No	Reference			Reference		
Chronic conditions						
Yes	2.50 (1.50–4.20)			5.18 (0.79–7.91)		
No	Reference			Reference		
Self-perceived health status						
Very good/good	Reference	Reference		Reference	Reference	
Fair	2.80 (1.82–4.30)	2.42 (1.52–3.84)	<0.001	2.90 (2.19–3.84)	2.43 (1.77–3.32)	<0.001
Very poor/poor	5.27 (3.38–8.24)	5.92 (3.59–9.75)	<0.001	5.96 (4.47–7.95)	5.82 (4.16–8.14)	<0.001

OR: odds ratio; ORa: odds ratio adjusted for all sociodemographic and health-related variables; CI 95%: 95% confidence interval; Rural residents: Hosmer–Lemeshow test for FL χ^2^ = 2.36, *p* = 0.94; Nagelkerke’s R^2^ Square for FL = 0.34; *p*-value < 0.001; Urban residents: Hosmer–Lemeshow test for FL χ^2^ = 11.41, *p* = 0.11; Nagelkerke’s R^2^ Square for FL = 0.34; *p*-value < 0.001.

**Table 4:** Association between PSS, sociodemographic factors and health-related determinants in rural and urban cancer patients (*n* = 2522).

Variables	Rural Residents						Urban Residents					
	Univariate Analysis			Multivariate Analysis			Univariate Analysis			Multivariate Analysis		
	B	β	*p*-Value	B	β	*p*-Value	B	β	*p*-Value	B	β	*p*-Value
Gender												
Men	Reference	Reference					Reference	Reference				
Women	0.71	0.65	0.28				0.20	0.01	0.62			
Age group												
<65 years old	Reference	Reference					Reference	Reference				
≥65 years old	0.09	0.005	0.89				−0.11	−0.07	0.78			
Educational attainment												
Without/Primary studies	−0.67	−0.04	0.32				−0.06	−0.01	0.98			
Secondary/University studies	Reference	Reference					Reference	Reference				
Marital status												
Married	2.47	0.14	<0.001	2.48	0.15	<0.001	2.48	0.15	<0.001	2.38	0.14	<0.001
Non-married	Reference	Reference		Reference	Reference		Reference	Reference		Reference	Reference	
Social class												
Upper	Reference	Reference					Reference	Reference				
Middle	0.13	0.008	0.90				−0.38	−0.02	0.47			
Lower	−0.28	−0.02	0.79				−1.02	−0.06	0.06			
Nationality												
Spanish	0.48	0.007	0.85				2.82	0.05	0.04	2.71	0.05	0.04
Foreigner	Reference	Reference					Reference	Reference		Reference	Reference	
Body Mass Index							0.19	0.01	0.64			
Normal weight	Reference	Reference					Reference	Reference				
Non-normal weight	−0.32	−0.02	0.62									
Hospitalization during the previous year												
Yes	0.71	0.76	<0.001				0.18	0.23	<0.001			
No	Reference						Reference	Reference				
Use of emergency services during the previous year												
Yes	0.18	0.67	<0.001				0.65	0.40	<0.001			
No	Reference						Reference	Reference				
Visits to specialist physician during the previous 4 weeks												
Yes	0.21	0.69	<0.001				0.18	0.25	<0.001			
No	Reference						Reference	Reference				
Visits to general practitioner during the previous 4 weeks												
Yes	0.14	0.65	<0001				0.17	0.39	<0.001			
No	Reference						Reference	Reference				
Smoking status												
Yes	−2.14	−0.08	0.03	−1.95	−0.07	0.04	−1.03	−0.05	0.05			
No	Reference	Reference		Reference	Reference		Reference	Reference				
Alcohol intake in the last year												
Yes	1.63	0.09	0.02				−0.20	−0.01	0.61			
No	Reference	Reference					Reference	Reference				
Chronic conditions												
Yes	0.63	0.03	0.48				1.54	0.07	<0.01	1.09	0.05	0.04
No	Reference	Reference					Reference	Reference		Reference	Reference	
Self-perceived health status												
Very good/good	Reference	Reference		Reference	Reference		Reference	Reference		Reference	Reference	
Fair	−1.21	−0.07	0.04	−1.22	−0.07	0.03	−0.98	−0.06	0.04	−0.88	−0.05	0.04
Very poor/poor	−2.91	−0.16	0.000	−3.07	−0.17	<0.001	−2.42	−0.13	<0.001	−2.28	−0.12	<0.001

B: Unstandardized coefficient; β: Standardized coefficient; Rural: Adjusted coefficient of determination (R^2^) = 21.33%, F = 8.44, *p* < 0.001; Urban: Adjusted coefficient of determination (R^2^) = 26.50%, F =14.48, *p* < 0.001.

## Data Availability

The data presented in this study are available as Supplementary Materials (File S1: Research data).

## Supplementary Materials

The following supporting information can be downloaded at: https://www.mdpi.com/article/10.3390/jcm11102742/s1, File S1: Research data.
